# Sampling Hyperpolarized Molecules Utilizing a 1 Tesla Permanent Magnetic Field

**DOI:** 10.1038/srep32846

**Published:** 2016-09-06

**Authors:** Sui Seng Tee, Valentina DiGialleonardo, Roozbeh Eskandari, Sangmoo Jeong, Kristin L. Granlund, Vesselin Miloushev, Alex J. Poot, Steven Truong, Julio A. Alvarez, Hannah N. Aldeborgh, Kayvan R. Keshari

**Affiliations:** 1 Department of Radiology and Molecular Pharmacology Program, Memorial Sloan Kettering Cancer Center, New York, NY 10065, USA; 2 Hunter College, New York, NY 10065, USA; 3 Weill Cornell Medical College, NY 10065, USA

## Abstract

Hyperpolarized magnetic resonance spectroscopy (HP MRS) using dynamic nuclear polarization (DNP) is a technique that has greatly enhanced the sensitivity of detecting ^13^C nuclei. However, the HP MRS polarization decays in the liquid state according to the spin-lattice relaxation time (T_1_) of the nucleus. Sampling of the signal also destroys polarization, resulting in a limited temporal ability to observe biologically interesting reactions. In this study, we demonstrate that sampling hyperpolarized signals using a permanent magnet at 1 Tesla (1T) is a simple and cost-effective method to increase T_1_s without sacrificing signal-to-noise. Biologically-relevant information may be obtained with a permanent magnet using enzyme solutions and in whole cells. Of significance, our findings indicate that changes in pyruvate metabolism can also be quantified in a xenograft model at this field strength.

Nuclear magnetic resonance (NMR) spectroscopy of biologically relevant nuclei, aside from protons, has long been limited by the lack of sensitivity. The development of hyperpolarization (HP) techniques has led to a dramatic increase in the signal-to-noise ratio in solution state. HP can be achieved by a number of methods including optical pumping^1^, para-hydrogen-induced polarization (PHIP)^2^,^3^ as well as dissolution dynamic nuclear polarization (DNP)^4^. Of these, DNP has received the most attention with a clinical trial recently completed^5^.

Regardless of how HP is achieved, the polarization decays in the liquid state according to the spin-lattice relaxation time (T_1_) of the nucleus. Sampling of the signal also uses polarization, resulting in a limited temporal ability to observe biologically interesting information if small flip angles are not employed. There have been several methods attempted to preserve nuclear polarization after dissolution. One approach involves converting polarization to a nuclear singlet-state order that can be stored and accessed. This can be accomplished through field cycling, continuous irradiation or chemical modification but none of these methods are compatible with biologically relevant imaging systems^6^. A more recent experiment utilized a ^13^C-labeled molecule with coupling constants that exceed the chemical shift difference demonstrated a modest increase in T_1_^7^. Currently, the singlet-state can only be accessed with a limited class of chemical structures and a more practical method of increasing the lifetime of polarization is by substituting exchangeable protons with deuterium (^2^H). This has been demonstrated with a number of different metabolites^8^,^9^,^10^, with reasonable increases in T_1_, although this method might be prohibitive due to the high costs involved in synthesizing deuterated substrates, limiting for screening large libraries of non-labeled compounds, as well as the potential for an isotope effect slowing biochemical kinetics^11^.

Many of the nuclear spins chosen for ^13^C isotopic enrichment and exploration tend to be carbonyl carbons since they are devoid of directly attached relaxation centers (protons) and are present in many biological systems^12^. Unlike in quaternary carbons, where T_1_ relaxation is dominated by dipole-dipole relaxation, carbonyl carbons additionally suffer from chemical shift anisotropy (CSA) or wobble induced relaxation. The T_1_ relaxation time under CSA is inversely proportional to the square of the external magnetic field strength (B_o_^2^). Therefore, in addition to the methods described to prolong the T_1_, sampling of the HP signal in a lower external magnetic field would result in prolonging the T_1_s of the molecules. To our knowledge, there have been two previous studies investigating the effects of external field strengths on T_1_. Chattergoon and colleagues investigated the field dependence of T_1_ for HP [1-^13^C] pyruvate using field-cycled relaxometry. From field strengths between 0.237 mT to 0.705 T, the authors observed a steady increase in T_1_^13^. Meanwhile, Mieville and colleagues investigated the R_1_ (equivalent to the reciprocal of T_1_), which reached a minimum at fields close to 1T^14^. Here, we hypothesize that sampling the HP signal using a permanent magnet at 1T will be a simple and cost effective way to significantly increase T_1_ values, regardless of the method of HP. The majority of MRI and NMR spectroscopy have utilized large, cryogenically cooled superconducting magnets to establish the static B_0_ field^15^. To the best of our knowledge, the use of a permanent, 1T spectrometer has yet to be demonstrated with hyperpolarized MR.

## Results

**Figure 1:**
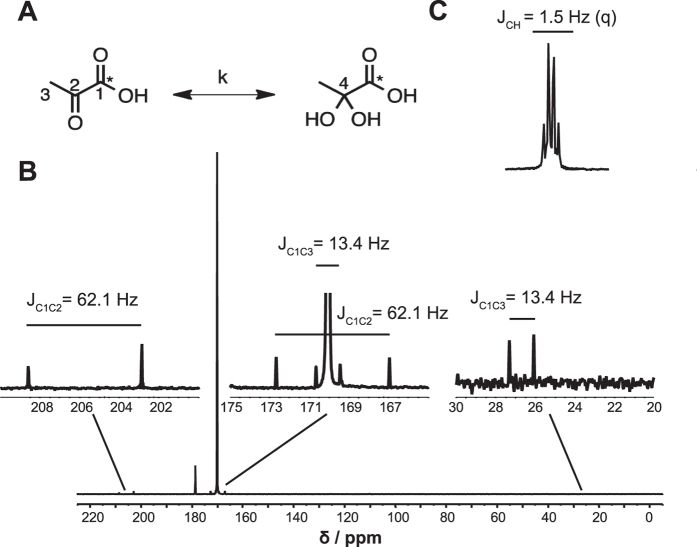
(**A**) Schematic of the spontaneous conversion of a solution containing 1.6 μmol of hyperpolarized [1-^13^C] pyruvate (in the active region of the coil) to [1-^13^C] pyruvate hydrate in a buffered solution at pH 7.4 at RT. This conversion can be observed in the first scan acquired using a 1T permanent magnet, with the scalar coupling (J coupling) of the different carbon functional groups easily observed. Coupling, between C1 and C2 of pyruvate results in a doublet separated by 62.1 Hz while coupling between C1 and C3 is separated by 13.4 Hz (**B**). Proton-carbon couplings can also be observed at this field strength (**C**), as evidenced by a quartet separated by 1.5 Hz.

We demonstrate that the shim achieved with a permanent magnet is satisfactory (Supp. Fig. S1). Additionally, with a pre-saturation pulse, the NMR signal derived from water can be suppressed to allow the visualization of biologically relevant compounds in such as choline (Supp. Fig. S2). Figure 1 demonstrates the clear distinction and measurement of the spin-spin coupling between the ^13^C-enriched carbon (99% ^13^C) of [1-^13^C] pyruvate and the adjacent natural abundance C2 and C3 carbons (1.1% ^13^C). Excellent signal-to-noise ratios (SNR) enable observation of these scalar couplings within the first scan of the experiment, allowing rapid determination of the chemical environment surrounding the labeled atom. Interestingly, the coupling between carbons and protons is also visible at this field strength by acquiring data without ^1^H decoupling. Carbons attached to adjacent protons displayed a distinctive 13.4 Hz doublet while a 62.1 Hz doublet was observed due to C1-C3 and C1-C2 coupling (Fig. 1B).

To demonstrate that sampling at 1T preserves the T_1_ of a variety of molecules, we proceeded to polarize and dissolve a range of molecules enriched at different functional groups. Table 1 summarizes the T_1_ of HP compounds that we have measured at 1T compared to literature values. There was an appreciable lengthening of the T_1_ times across a wide range of carbonyl containing chemical structures, including keto-acids and amino acids. As expected non-carbonyl carbons, or carbons with minimal CSA, did not have significantly increased T_1_s.

**Figure 2:**
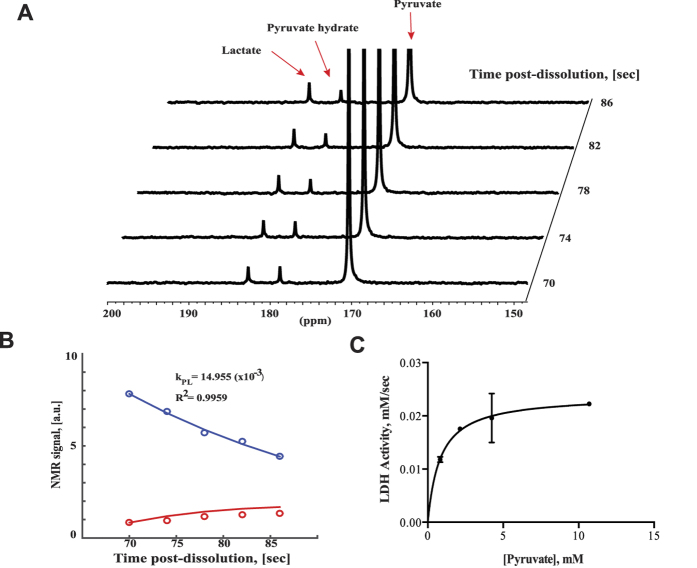
(**A**) Dynamic spectra of a solution 4 mM hyperpolarized pyruvate and 0.4 U of LDH enzyme sampled every 4 s with a 10° flip angle. Visible resonances are pyruvate (171 ppm), pyruvate hydrate (178 ppm) and lactate (182 ppm). (**B**) Integrals of the resonances were fit to a model to extract rate constants, where pyruvate (blue) decays over time while lactate (red) increases. (**C**) Different pyruvate concentrations (0.82–10.7 mM, n = 3 each concentration) were used to determine the kinetics of LDH.

To demonstrate the feasibility of the system to quantify biologically-relevant reactions, we measured the kinetics of lactate dehydrogenase (LDH) that catalyzes the conversion of pyruvate to lactate. The use of a low-field permanent magnet enabled the close positioning of the spectrometer to the hyperpolarizer, enabling multiple measurements from a single dissolution. Figure 2A depicts a representative experiment where 4 mM of hyperpolarized pyruvate was injected into a 5 mm NMR tube containing 0.4 U of LDH. The data were fit to a model of interconversion from pyruvate to lactate^22^ to produce a flux rate, k_PL_ (Fig. 2B). By repeating the experiment using different concentrations of pyruvate, we were able to quantify the kinetics of LDH, with a V_max_ value of 0.024 mM/sec and a K_m_ value of 0.82 mM (Fig. 2C). The K_m_ value obtained approximates literature values^23^, suggesting that hyperpolarized MRS might be an alternative method to accurately quantify enzyme kinetics where no other assays are available. The majority of HP experiments in perfused bioreactors have been performed on high field NMR systems^22^,^24^ (e.g. 11.7–14.1T). These experiments require a constant flow of growth media, gaseous exchange and temperature control. Integration of these requirements onto existing NMR systems can be cumbersome and risks damaging expensive, superconducting systems. In contrast, a permanent 1T magnet has a small footprint, allowing perfusion and gaseous exchange platforms to be built around it. In an experiment of alginate-encapsulated PC3 prostate cancer cells, an injection of hyperpolarized [1-^13^C] pyruvate resulted in the production of [1-^13^C] lactate but with a quarter of the number of cells used in previous publications^25^ (Supp. Fig. S3A). We were also able to detect peaks of total choline (tCho) and lactate, using proton spectroscopy after the application of a water saturation pulse (Supp. Fig. S3B).

**Figure 3:**
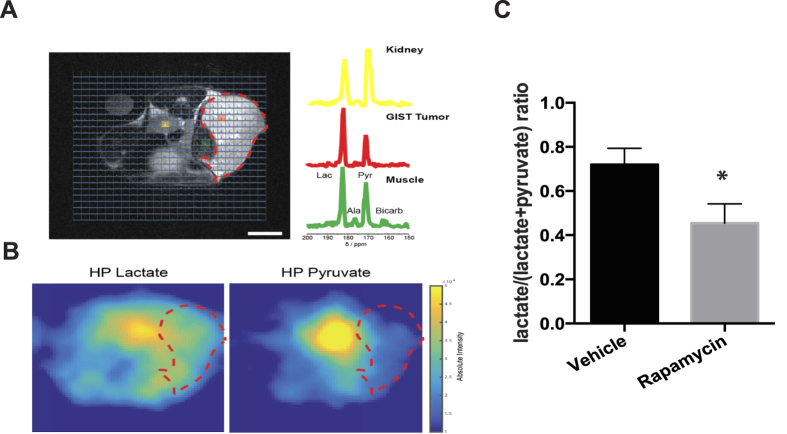
(**A**) T_2_-weighted image of a GIST xenograft injected with 0.05 mg/kg hyperpolarized [1-^13^C] pyruvate. Metabolites in kidney (yellow) and muscle (green) are markedly different from tumor (red) as evidenced by the higher levels of lactate produced within the tumor. (**B**) False-color images across the image section reveals higher levels of lactate in the tumor region. (**C**) Treatment with a cancer drug, rapamycin (10 mg/kg, 24 hr before imaging), results in lower levels of lactate production after hyperpolarized pyruvate injection.

We went on to demonstrate the feasibility of acquiring spectroscopic imaging data from hyperpolarized substrates in live animals. A subcutaneous GIST-T1 sarcoma was subcutaneously xenografted on a mouse and injected with 0.05 mg/kg of [1-^13^C] pyruvate. While the resultant [1-^13^C] lactate was visible in regions of the muscle and kidney, this peak was evidently larger in tumor regions (Fig. 3A). This spectroscopic data can also be used to create false-color images using the same 1T permanent magnet, allowing localization of regions of high lactate production when superimposed on regular anatomical images (Fig. 3B). To demonstrate that we were able to detect cancer treatment response, tumors were treated with rapamycin, a potent inhibitor of the mammalian target of rapamycin (mTOR). Rapamycin treatment has already been shown to decrease glycolytic flux to lactate and can be detected using radioactive PET imaging^26^. In Fig. 3D, we demonstrate that by using our small animal imaging setup, a significant decrease of 38% (p-value <0.01) of hyperpolarized lactate was observed 24 hrs post-rapamycin treatment.

## Discussion

HP MRI has been informative in many fields including tumor metabolism^27^, cardiac biology^28^ as well as inflammation^29^. There has been much excitement over the potential of this imaging modality and a simple, cost-effective method to sample hyperpolarized molecules will ensure that this technology can be adopted more extensively. In a literature search on Pubmed with the terms ‘hyperpolarized’ and ‘dynamic nuclear polarization’, we assessed 185 publications dating back to 2002 and uncovered no instances of HP experiments performed on permanent magnets with the lowest field strength recorded was a 2.4 T super-conducting, small animal imaging system^30^. Therefore, we believe that this study represents the first systematic demonstration that HP MRI can be reliably performed using permanent magnets at low field. While the drift of a permanent magnet can be significant, the availability of field locks ensures this potential problem will be mitigated. Additionally, the lifetime of the HP experiment is in the order of seconds to minutes, ensuring that intra-experimental drift will be negligible. Sampling at lower field strengths will result in significant homonuclear ^13^C couplings resulting in the observation of multiplets in the spectra. This might complicate quantification, but we also believe that this might be an advantage in elucidating molecules of unknown structure. We also observe intensity asymmetries in the doublet centered at 27 and 206 ppm. We attribute this asymmetry to differential polarization of the coupled ^13^C nuclei, consistent with observations made previously^31^. While there has been much excitement for the translation of HP MRI to the clinic, the majority of hospital scanners operate at 1.5T^32^. If HP MRI is to be widely tested in multi-center human trials, an understanding of the behavior of molecules at clinically-relevant field strengths would be essential. There are a number of other benefits from using a low-field system, including less susceptibility effects, ease of use as well as the ability to rapidly integrate MR with other imaging modalities such as PET. We believe that HP experiments *in vivo* will especially benefit from the lengthened T_1_s at 1T because the longitudinal relaxation time of HP molecules have been shown to be shorter *in vivo* as compared to in solution. Longer T1s will also enable the simultaneous measurement of a multi-enzyme cascade, where the fate of the polarized metabolite is known, from a single dissolution. The effect of T_1_ lengthening is by no means a unique property of hyperpolarized measurements at 1T, nor do they represent the maximum T_1_ values obtainable. However, the availability of benchtop spectrometers at this field strength and ease of use adds to attractiveness of sampling hyperpolarized molecules at 1T. Additionally, the majority of hyperpolarized metabolites are small molecules with correlation times that will benefit from sampling at lower field strengths^33^. Furthermore, co-polarization studies can also be performed to measure kinetics of several different metabolites being acted upon by different enzymes. Previous studies polarizing multiple compunds simultaneously utilized a 3T magnet^34^ and we believe similar, quantifiable results should be obtainable using a 1T permanent magnet. In summary, we believe sampling hyperpolarized molecules at low-field has both practical benefits besides providing novel information.

## Methods

### HP molecules

Unless otherwise indicated, all chemicals and solvents were purchased from Sigma-Aldrich (St. Louis, MO). 13C metabolites were prepared for HP according to published reports: [1-13C] pyruvate^24^, [1-13C] lactate^35^, [13C,15N2] urea^36^, [1-13C] dehydroascorbate and [1-13C] ascorbate^21^. All metabolites were HP using a prototype SpinLab (General Electric, Niskayuna, New York, USA) for approximately 90 min before dissolving with appropriate buffers as described in previous publications.

### NMR and T_1_ determination

NMR studies were performed on a 1 Tesla Magritek Spectrometer (Magritek, San Diego, CA) using a 5 mm ^1^H/^13^C coil. The dissolution process, transfer of solution and transit to the spectrometer was approximately 20 s and this was accounted for in all T_1_ calculations. All transfer was performed in the presence of a permanent magnet to avoid depolarization. For the acquisition of HP spectra in solution, a repetition time (TR) of 3 s was applied for a total of at least 64 scans. For T_1_ measurements, HP solutions were placed into a pre-warmed NMR tube and a series of spectra was collected with 3 s temporal resolution. These spectra were then fit to a mono-exponential decay function, correcting for flip angle, to determine the spin-lattice relaxation time as previously described^8^.

### Cell culture, enzyme and bioreactor experiments

#### PC3 prostate cancer and GIST-T1 sarcoma cells were grown under standard conditions

For experiments with LDH-A pure enzyme, commercial rabbit LDH-A (Sigma-Aldrich, St. Louis, MO) was diluted in Tris-HCL buffer, pH 7.4. For kinetic experiments, 1 U enzyme was used with NADH concentrations of 1 mM. The hyperpolarized pyruvate-to-lactate flux was quantified using a previously published model^22^.

For bioreactor experiments, 92 hr before imaging, cells were trypsinized, pelleted and resuspended in a sodium alginate solution in HBSS at a concentration of 1 × 10^8 ^cells/ml. After thorough mixing, the cell suspension was extruded through a 23 G angiocatheter to beads of approximately 500 μm in diameter. On the day of imaging, beads were inserted into a custom-built 5 mm NMR-compatible bioreactor. HP [1-^13^C] pyruvic acid was dissolved and injected into the perfusion system. Spectra were acquired using a 10° excitation pulse every 10 seconds. A section of the bead was fixed in 10% neutral buffered formalin for histology while the rest dissociated for trypan blue exclusion and cell counting.

For water suppression, a presaturation pulse at 18.3 Hz at an amplitude of −25 dB and 2000 ms duration was applied at 1000 ms repetition time, 11 us pulse length, 20 us acquisition delay and a gradient amplitude of 7000.

#### *In vivo* experiments

The animal portion of this study was performed in accordance with a protocol approved by the Institutional Animal Care and Use Committee (IACUC). 5 × 10^6^ GIST-T1 cells were trypsinized and resuspended in a 1:1 solution of complete media: Matrigel. A total of 6 animals were subcutaneously injected on the flank of NOD/SCID mice. Mice were imaged utilizing a hybrid 1T MR/PET system (Mediso, USA) equipped with a ^1^H/^13^C dual tune RF probe. ^1^H T_2_-weighted fast spin echo (FSE) acquisition, T_E_/T_R_ = 12.4/671 ms, 0.3125 × 0.3125 mm in plane resolution and 2 mm slice thickness images were used to denote the anatomy and define the tumor region of interest as well as perform volume measurements. Tumors of approximately 1 cm^3^ were visible after approximately 4 weeks. 24 hrs before the HP studies the 6 animals were randomly divided in vehicle (6% DMSO) and rapamycin (15 mg/kg) treated group. For HP ^13^C Imaging, post-infusion of HP [1-^13^C] pyruvate (0.1 mg/g pH = 7.4 over 10 s) either a slab dynamic ^13^C MR spectra (10° slab excitation every 3 s, slab thickness = 1 cm) or a 2D ^13^C magnetic resonance spectroscopic imaging (MRSI, delay = 20 s, 20° constant flip angle, P_E_ = 12 × 12, in-plane 2.5 × 2.5 mm resolution, slab thickness = 1.3 cm) sequence was employed.

## Additional Information

**How to cite this article**: Tee, S. S. *et al*. Sampling Hyperpolarized Molecules Utilizing a 1 Tesla Permanent Magnetic Field. *Sci. Rep.* **6**, 32846; doi: 10.1038/srep32846 (2016).

## Supplementary Material

Supplementary Information

## Figures and Tables

**Table 1:** Apparent spin-lattice relaxation times (T_1_) for various hyperpolarized molecules at 1 Tesla compared to previously published values.

Compound	HP T_1_ at 1T	Literature T_1_
[1-^13^C] pyruvate	71.3 ± 1.8s	40.0 s (9.4T)^16^
[1-^13^C] glutamate	66.5 ± 6.4s	33.9 s (9.4T)^17^
[1-^13^C] oxalate	66.7 ± 7.0 s	n/a
[1-^13^C] lactate	42.9 ± 4.2 s	33 s (14.1T)^18^
[^13^C-^15^N] urea	50.7 ± 1.8 s	44 s (3T)^19^
[1-^13^C] methionine	47.6 ± 2.8 s	17 s (9.4T)^20^
[1-^13^C] dehydroascorbate	75.1 ± 3.1 s	57 s (3T)^21^

All metabolites were dissolved in a buffered solution at pH = 7.4 and measurements performed at 25 °C.
