# Supplementary material for: Sampling Hyperpolarized Molecules Utilizing a 1 Tesla Permanent Magnetic Field
Source: Sci Rep. 2016 Sep 6;6:32846. doi: 10.1038/srep32846 (PMC5011774; doi:10.1038/srep32846)
Supplement: Supplementary Information [file srep32846-s1.pdf]

Supplementary Information

Sampling Hyperpolarized Molecules Utilizing a 1 Tesla Permanent Magnetic Field

Sui Seng Tee‡[1], Valentina DiGialleonardo‡[1], Roozbeh Eskandari[1], Sangmoo Jeong[1], Kristin L. Granlund[1], Vesselin Miloushev[1], Alex J. Poot[1], Steven Truong[2], Julio A. Alvarez[3], Hannah N. Aldeborgh[1], Kayvan R. Keshari[1,3]*

[1]Department of Radiology and Molecular Pharmacology Program, Memorial Sloan Kettering Cancer Center, New York, NY 10065, USA

[2]Hunter College, New York, NY 10065, USA

[3]Weill Cornell Medical College, NY 10065, USA

Supplementary Figures

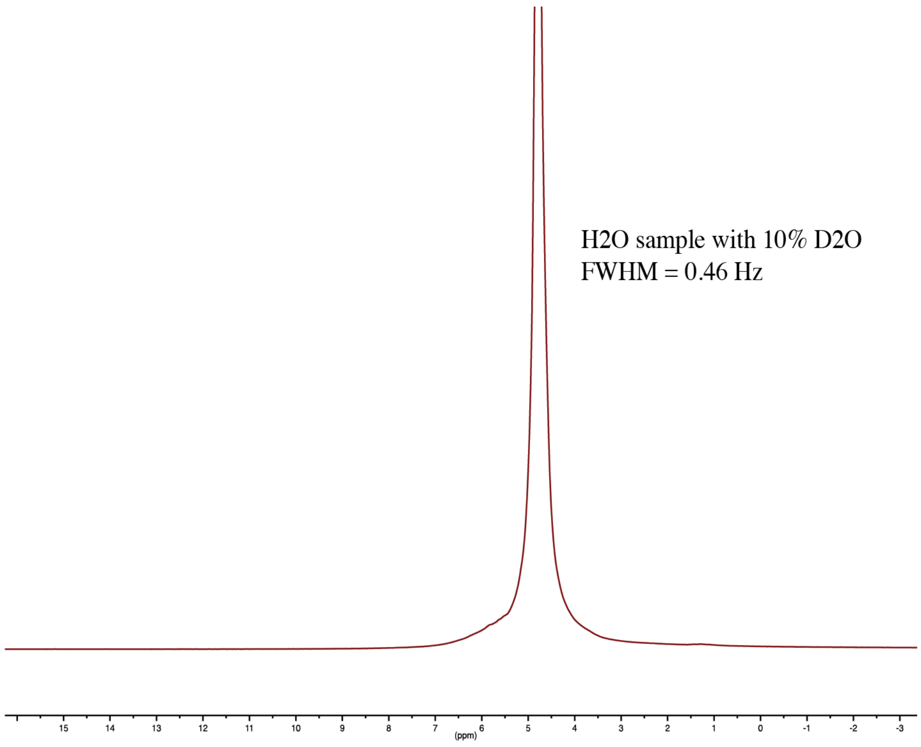

Supp Fig S1: Representative spectrum of a 90:10 H20:D20 sample. The external magnetic field of the spectrometer with a 1 Tesla permanent magnet consistently produces shim values of < 1.0 Hz.

(A) (B)

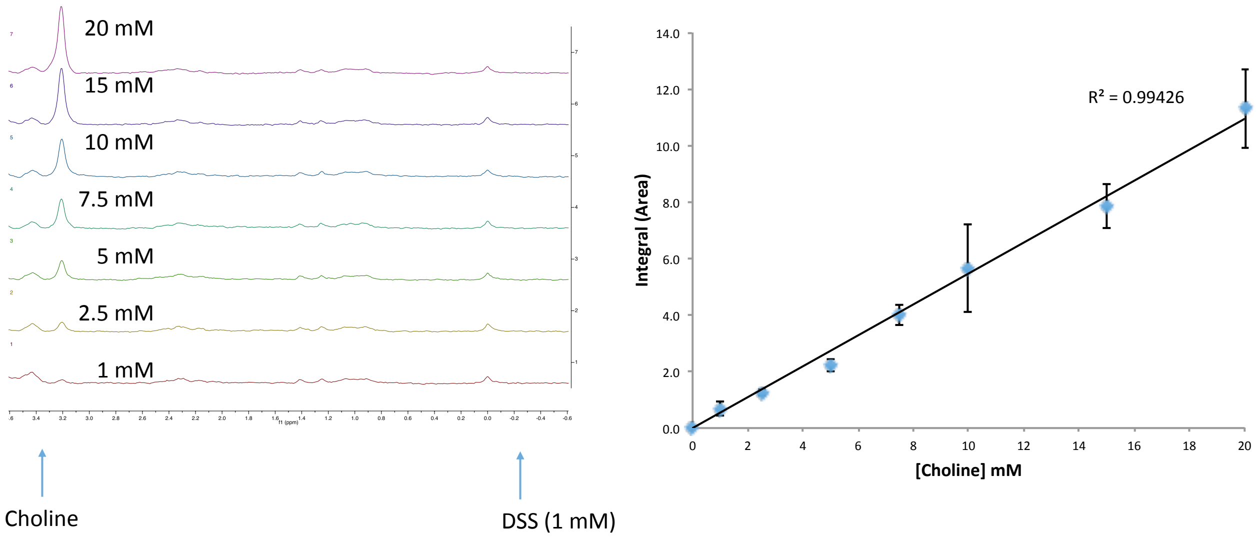

Supp Fig S2: (A) Representative spectra of known choline concentrations spiked in DMEM cell culture media with 1mM DSS as a chemical shift indicator. Spectra were acquired using a presaturation pulse (details in *Experimental Section*). (B) Choline peak integrals are linear over various concentrations of choline in complete cell culture media (0.1 to 20 mM).

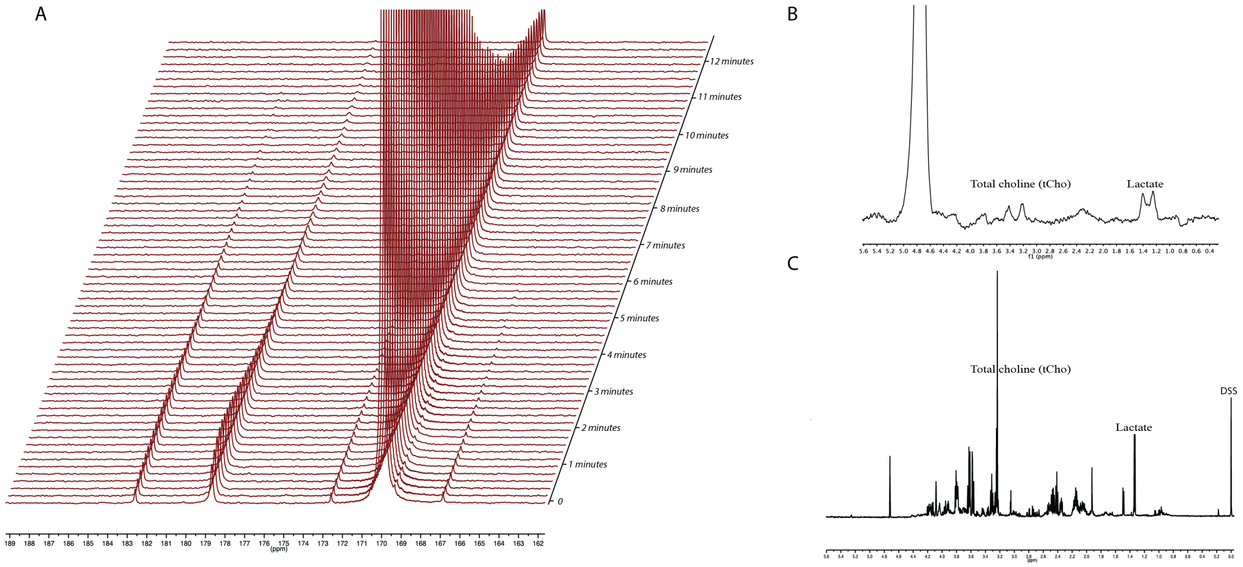

Supp Fig S3: (A) In a perfused bioreactor experiment, PC3 cells were encapsulated in alginate and these 3D cultures were able to convert [1-13C] pyruvate to lactate (B) Proton spectroscopy of the same alginate beads allows the visualization of intracellular choline (tCho) and lactate after application of a water saturation pulse (C) Confirmation of the high concentrations of tCho in these cells by proton spectroscopy of cells after methanol extraction using a 600 Mhz research magnet.

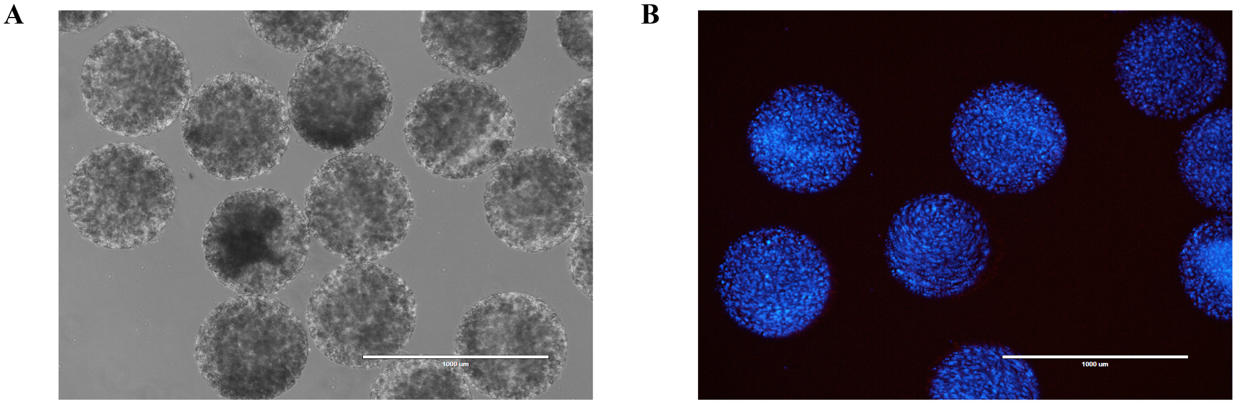

Supp Fig S4: (A) Phase-contrast microscopy images of PC3 cells encapsulated in sodium alginate and (B) the same cells stained with DAPI to visualize nuclei. Scale bar represents 1000um

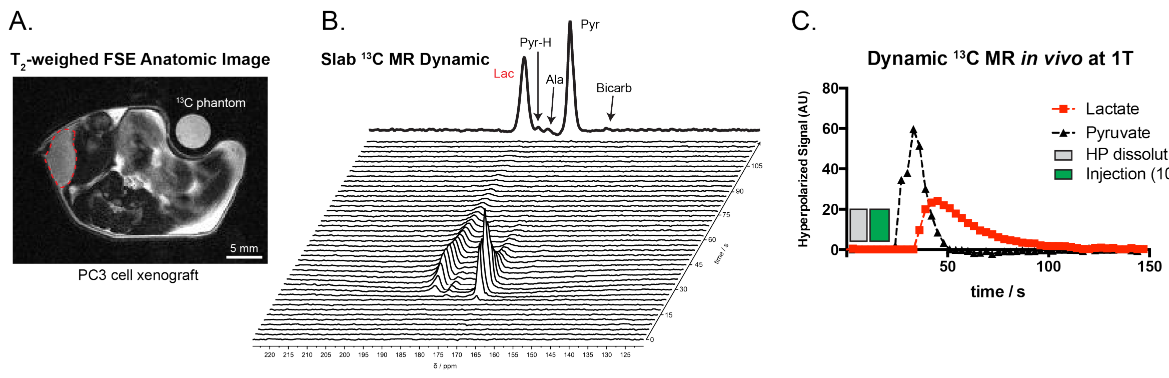

Figure S5: (A) T2-weighted fast-spin echo (FSE) image of a mouse bearing PC3 prostate cancer cell xenograft that was (B) subsequently injected with 0.05mg/kg hyperpolarized [1-13C] pyruvate. Visible peaks are annotated above and the magnitude of the pyruvate and lactate signals were quantified over time (C)

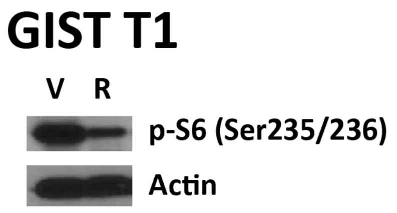

Supp Fig S6: Western blot of tumors treated with either DMSO (V) or 100mg/kg rapamycin for 24 hr (R) probed with primary antibody for phospho-S6 at Ser235/236 and actin as a loading control
